# Editorial: Genetics and Genomics of Red Blood Cells

**DOI:** 10.3389/fphys.2021.822156

**Published:** 2022-01-07

**Authors:** Achille Iolascon, Roberta Russo, Immacolata Andolfo

**Affiliations:** ^1^Dipartimento di Medicina Molecolare e Biotecnologie Mediche, Università di Napoli Federico II, Naples, Italy; ^2^CEINGE Biotecnologie Avanzate, Naples, Italy

**Keywords:** hereditary anemias, next-generation sequencing, transcriptomics, genetic diagnosis, genetic variants, transgenic animal model

The current Research Topic focused on the implementation of next-generation sequencing (NGS) applications in the field of genetics, genomics, and transcriptomics of red blood cell defects. This is an important issue in the current scientific scenario either in the clinical/diagnostic or in research settings. Indeed, the widespread use of these technologies has already modified the approaches to diagnosis and research for red blood cell disorders (Russo et al., 2020).

In their review, Achour et al. discussed the role of NGS in both screening and pre- and post-natal diagnostics of the hemoglobinopathies. Based on the analysis of literature data, they concluded that NGS has an added value in large-scale high throughput carrier screening and in the complex cases that are often missed using the traditional approach. Most of thalassemias and hemoglobinopathies can be diagnosed using routine analysis that include a combined approach based on the evaluation of complete blood count, hemoglobin separation, and first-generation DNA sequencing (Taher et al., 2021). Nevertheless, Achour et al. concluded that NGS-based technology is not likely to replace existing methods but can be a useful additional tool in the diagnostic strategy of these conditions. This is mainly true for large-scale genetic screening, in the discovery of novel pathogenic mechanisms, in a better understanding of the erythroid-specific beta-gene regulation of expression, and in an improved establishment of genotype-phenotype correlation.

One of the major advantages of the NGS approach is the identification of both the polygenic conditions and the modifier variants associated with causative mutations (Andolfo et al., 2021). Several NGS-based studies identified genetic polymorphisms as phenotype modifiers with a mild effect on disease expression under a “multifactorial-like” model. Indeed, under this model, the disease expression depends on the effects of several genetic variants located in different modifier genes that, by themselves, only explain a small proportion of the variability but interact both with one another and with environmental factors (Rund and Fucharoen, 2008; Andolfo et al., 2018, 2019).

In their study, Lionetti et al. investigated eight clinically relevant single nucleotide polymorphisms (SNPs) within *NR3C1* gene, encoding the glucocorticoid receptor, in patients with Diamond Blackfan Anemia (DBA) (Da Costa et al., 2020). *NR3C1* is a highly polymorphic locus harboring numerous SNPs associated with variability of manifestation and/or response to glucocorticoids in human diseases. Of note, ~60–80% of DBA patients improve anemia and became transfusion independent upon treatment with glucocorticoids. Two out eight SNPs of *NR3C1* regulatory regions, rs6196 and rs860457, were enriched in patients manifesting the disease before 4 months of age, suggesting that these variants may regulate the response of erythroid cells to glucocorticoids during ontogeny. This hypothesis was supported by phosphoproteomic profiling of erythroid cells expanded *ex vivo* indicating that glucocorticoids activate a ribosomal signature in cells from cord blood but not in those from adult blood.

From the research perspective, the use of NGS applications allowed both identifying new causative genes associated to red blood cell defects as well as understanding the pathogenic mechanisms underlying these disorders. Within this Research Topic, the study by Karaosmanoglu et al. investigated the transcriptomic profile of three different cell types of bone marrow resident cells isolated from patients affected by DBA related to *RPS19* and *CECR1* mutations. The authors observed increased expression of genes associated with both cellular stress and immune response. Interestingly, they also suggested that the gene expression profile reflecting cellular stress and cytokine response in proerythroblasts may be associated with increased inflammation in the bone marrow niche. This observation agrees with previous studies suggesting a specific contribution of TNF-α in the inhibition of erythroid differentiation and in the pathogenesis of ribosomal stress observed in DBA.

The importance and the advantages of NGS are easily understandable. However, despite the extensive employment of this technique in routine laboratory practice, some considerations on its limitations and/or disadvantages should be done. A major limitation of NGS genome analysis remains the data processing steps and the assessment of the pathogenicity of identified genetic variants (Lincoln et al., 2021). This latter may be improved by generating engineered cellular or animal models to study the pathogenesis of the diseases.

The study by Noy-Lotan et al. investigated the role of Codanin-1 in erythropoiesis. Codanin-1 is the protein product of *CDAN1*, the main causative gene of congenital dyserythropoietic anemia type I (CDA I) (Roy and Babbs, 2019; Iolascon et al., 2020). Codanin-1 is a cell-cycle regulated protein with high similarity to CNOT1, a conserved protein that serves as a scaffold for proteins involved in mRNA stability and transcriptional control (Swickley et al., 2020). Although different studies have clarified some biological function of Codanin-1, its cellular functions and the erythroid specificity of the phenotype remain elusive. To shed more light on the specific role of Codanin-1 in erythropoiesis, and to circumvent the early fatality caused by a complete knockout of Cdan1, the authors generated a Cdan1 erythroid conditional knockout mouse model by crossing Cdan1^*flox*/*flox*^ mice with a transgenic mouse strain that expresses the Cre-recombinase under regulation of the endogenous erythropoietin receptor promoter. The resulting Cdan^Δ*Ery*^ mice die *in-utero* at mid-gestation from severe anemia with very low numbers of circulating erythroblast. Of note Cdan^Δ*Ery*^ model displays severe aberrations of primitive erythropoiesis and erythroblasts exhibit the pathognomonic morphological features described in individuals with CDA I, suggesting that CDAN1 is required for primitive erythropoiesis.

This collection of articles related to the study of genetics, genomics, and transcriptomics of red blood cell defect highlighted the actual and future direction of the diagnosis and research in this field. Although these conditions are rare, the study of their pathogenetic mechanism could shed light on new mechanisms that can be shared with other conditions affecting larger populations.

## Author Contributions

AI, RR, and IA wrote the manuscript. All authors contributed to the article and approved the submitted version.

## Conflict of Interest

The authors declare that the research was conducted in the absence of any commercial or financial relationships that could be construed as a potential conflict of interest.

## Publisher's Note

All claims expressed in this article are solely those of the authors and do not necessarily represent those of their affiliated organizations, or those of the publisher, the editors and the reviewers. Any product that may be evaluated in this article, or claim that may be made by its manufacturer, is not guaranteed or endorsed by the publisher.
